# Alterations in Intrinsic and Synaptic Properties of Hippocampal CA1 VIP Interneurons During Aging

**DOI:** 10.3389/fncel.2020.554405

**Published:** 2020-10-14

**Authors:** Ruggiero Francavilla, Alexandre Guet-McCreight, Sona Amalyan, Chin Wai Hui, Dimitry Topolnik, Félix Michaud, Beatrice Marino, Marie-Ève Tremblay, Frances K. Skinner, Lisa Topolnik

**Affiliations:** ^1^Department of Biochemistry, Microbiology and Bioinformatics, Faculty of Science and Engineering, Université Laval, Québec, QC, Canada; ^2^Neuroscience Axis, Centre Hospitalier Universitaire (CHU) de Québec Research Center – Université Laval, Québec, QC, Canada; ^3^Krembil Research Institute, University Health Network, University of Toronto, Toronto, ON, Canada; ^4^Department of Molecular Medicine, Faculty of Medicine, Université Laval, Québec, QC, Canada; ^5^Division of Medical Sciences, University of Victoria, Victoria, BC, Canada; ^6^Department of Physiology, University of Toronto, Toronto, ON, Canada; ^7^Departments of Medicine (Neurology) and Physiology, University of Toronto, Toronto, ON, Canada

**Keywords:** circuit disinhibition, VIP, action potential, synapse, hippocampus, aging, calretinin

## Abstract

Learning and memory deficits are hallmarks of the aging brain, with cortical neuronal circuits representing the main target in cognitive deterioration. While GABAergic inhibitory and disinhibitory circuits are critical in supporting cognitive processes, their roles in age-related cognitive decline remain largely unknown. Here, we examined the morphological and physiological properties of the hippocampal CA1 vasoactive intestinal peptide/calretinin-expressing (VIP+/CR+) type 3 interneuron-specific (I-S3) cells across mouse lifespan. Our data showed that while the number and morphological features of I-S3 cells remained unchanged, their firing and synaptic properties were significantly altered in old animals. In particular, the action potential duration and the level of steady-state depolarization were significantly increased in old animals in parallel with a significant decrease in the maximal firing frequency. Reducing the fast-delayed rectifier potassium or transient sodium conductances in I-S3 cell computational models could reproduce the age-related changes in I-S3 cell firing properties. However, experimental data revealed no difference in the activation properties of the Kv3.1 and A-type potassium currents, indicating that transient sodium together with other ion conductances may be responsible for the observed phenomena. Furthermore, I-S3 cells in aged mice received a stronger inhibitory drive due to concomitant increase in the amplitude and frequency of spontaneous inhibitory currents. These age-associated changes in the I-S3 cell properties occurred in parallel with an increased inhibition of their target interneurons and were associated with spatial memory deficits and increased anxiety. Taken together, these data indicate that VIP+/CR+ interneurons responsible for local circuit disinhibition survive during aging but exhibit significantly altered physiological properties, which may result in the increased inhibition of hippocampal interneurons and distorted mnemonic functions.

## Introduction

Aging is an inevitable and extremely complex physiological process often associated with a progressive deterioration of brain functions (Peters, 2006). Cognitive decline and memory deficits are considered hallmarks of the aging brain, with cortical circuits being affected the most during age-dependent functional decline (Murman, 2015). In particular, significant age-related structural and functional changes have been consistently reported in the human and rodent hippocampus (Pyapali and Turner, 1996; Rosenzweig and Barnes, 2003; Markham et al., 2005; Bamidis et al., 2014; Rizzo et al., 2014). Specifically, it was reported that while hippocampal pyramidal cells (PCs) survive in aging, their intrinsic and synaptic excitability is altered (Barnes, 1994; Moyer and Disterhoft, 1994; Rapp and Gallagher, 1996; Moyer et al., 2000; Power et al., 2002). For example, different studies have shown an age-related increase in the action potential (AP) threshold and duration, enhanced after hyperpolarization and a greater spike frequency adaptation in rodent CA1 PCs, revealing altered intrinsic excitability (Potier et al., 1992, 1993; Moyer et al., 2000; Power et al., 2002; Randall et al., 2012). In addition, hippocampal CA1 PCs in aged rodents show significantly reduced synaptic inhibition (Potier et al., 2006; Stanley et al., 2012) and, likely, enhanced excitation (El-Hayek et al., 2013). Specifically, the main excitatory input arriving to the CA1 from the hippocampal CA3 region shows abnormal activation with seizure-like electrical patterns in aged rodents (Wilson et al., 2005, 2006; Koh et al., 2010; El-Hayek et al., 2013). Interestingly, CA3 hyperactivity has been also reported in patients with the amnestic mild cognitive impairment (aMCI) and was among the risk factors for the development of Alzheimer’s disease (Bakker et al., 2012). Moreover, using a low dose of antiepileptic drug was beneficial in animal model and patients’ studies (Koh et al., 2010; Bakker et al., 2012), revealing imbalanced network activity as a primary mechanism for aMCI during aging.

The hippocampal CA1 network is composed of a large diversity of GABAergic inhibitory interneurons, which generate specific patterns of oscillatory activity playing a crucial role in different mnemonic processes (Somogyi et al., 1998). Quantitative anatomical studies showed that, compared to PCs, the populations of GABAergic interneurons are more vulnerable during aging. In particular, a significant reduction in the number of hippocampal CA1 somatostatin (SST+) but not parvalbumin (PV+)-expressing interneurons has been reported in aged rats (Stanley et al., 2012). The age-related changes in other interneuron types, including the cholecystokinin (CCK+)-, the neuropeptide Y (NPY+)- and the vasoactive intestinal peptide (VIP+)- or calretinin (CR)-expressing cells remain largely unknown.

Over the last several years, the disinhibitory circuits formed by the VIP+/CR+ interneuron-specific (I-S) interneurons that innervate GABAergic cells selectively (Acsády et al., 1996a,b; Gulyas et al., 1996) and can set a balance between network excitation and inhibition, have captured the attention of neuroscientists (Guet-McCreight et al., 2020). Despite their important role in coordinating hippocampal inhibition during spatial learning and goal-oriented behavior (Magnin et al., 2019; Turi et al., 2019), very little is currently known about the survival and functioning of I-S interneurons during aging. Here, we addressed this question by focusing on the type 3 VIP+/CR+ interneuron-specific (I-S3) cells that target different types of CA1 stratum oriens/alveus (O/A) interneurons and control both the perisomatic and dendritic inhibition converging onto CA1 PCs (Acsády et al., 1996b; Chamberland et al., 2010; Tyan et al., 2014; Francavilla et al., 2015). We provide evidence that I-S3 cells survive and preserve their morphology during aging in mice. However, the physiological properties of these cells undergo specific modifications over the course of aging. Thus, this study provides new evidence to support the functional remodeling of disinhibitory circuits in the aged hippocampus, leading to a compensatory potential reorganization of inhibition of the hippocampal CA1 interneurons, which may have an important impact on CA1 network activity and related mnemonic processes.

## Materials and Methods

### Mouse Line

**FIGURE 1:**
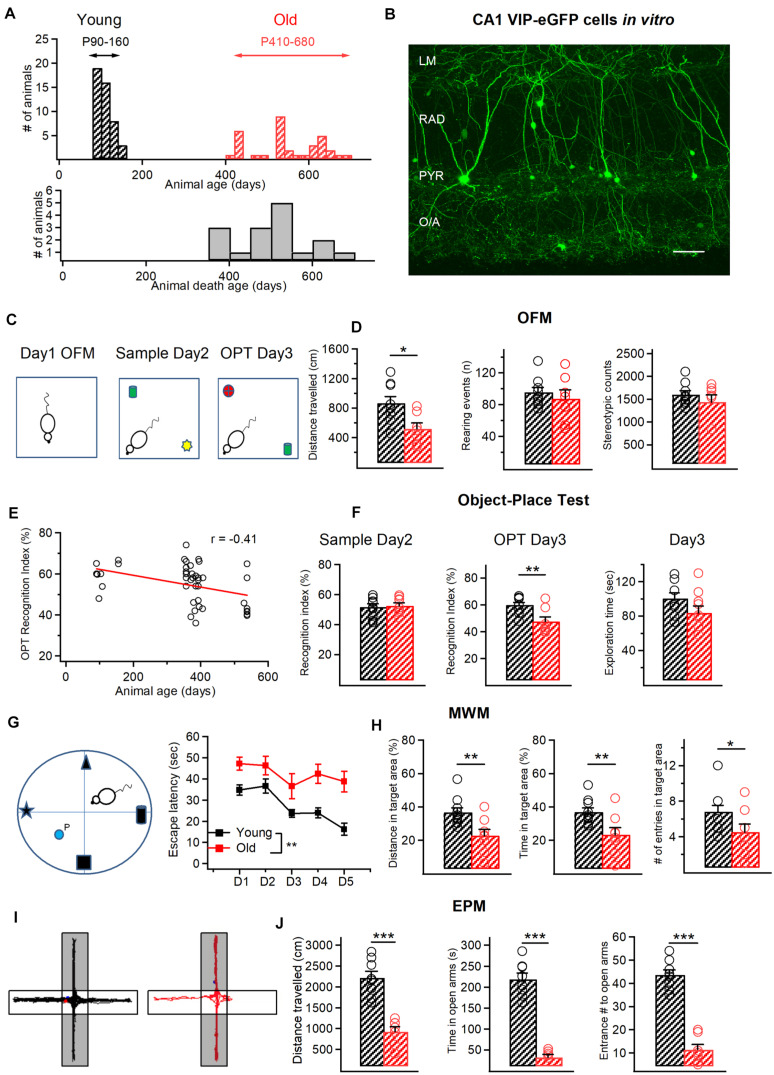
Age-related behavioralchanges in VIP-eGFP mice. **(A)** Top, distribution of the age of animals used in this study with two experimental groups: young (P90–150) and old (P420–680). Bottom, cumulative histogram indicating the age of death of VIP-eGFP mice (days). **(B)** Two-photon image of the CA1 area from an acute hippocampal slice (300 μm) of a VIP-eGFP mouse showing the location of VIP+ cells in different layers of CA1 area. Scale bar: 100 μm. **(C)** Schematics of the open field maze (OFM) and the object place test (OPT) with open field arena exploration on day 1 (left), sample phase (middle) with object A (green) and object B (yellow) on day 2, and test phase (right) with object C (red) and object A (green) on day 3. **(D)** Summary data for two groups of animals (young–black, old–red) showing a significant decrease in distance traveled with aging (*p* < 0.05, *t-*test, left), and no change in rearing events (middle) and stereotypic counts (right) during OFM test. **(E)** Negative correlation between the OPT recognition index (RI) and the mouse age (*R* = –0.41, *p* < 0.05, Spearman correlation). **(F)** Summary data showing no change in the RI during the sample phase (left) and the exploration time spent with the 2 objects on day 3 (right), but a significant decrease in the RI in the object-place test (*p* < 0.01, unpaired *t-*test, middle). **(G)** Schematics of the Morris Water Maze (MWM) test with four different spatial cues and a hidden platform (P) (left) and the escape latency from day 1 (D1) to Day 5 (D5) of training for young (black) and old (red) mice. **(H)** Summary data for two groups of animals (young–black, old–red) showing a significant reduction in the distance traveled (*p* < 0.01, *t*-test, left) and time (*p* < 0.01, *t*-test, middle) within the target area and the number of entries to the target area (*p* < 0.05, *t*-test, right) in old mice. **(I)** Representative activity tracks for young (left) and old mice (right) showing decreased activity for old mice in the open arms of the elevated plus maze (EPM). **(J)** Summary data showing that old animals explored less (*p* < 0.001, unpaired *t*-test, left), spent less time (*p* < 0.001, unpaired *t*-test, middle) and entered less frequently (*p* < 0.001, unpaired *t-*test, right) in the open arms of the EPM. *p < 0.05, **p < 0.01, ***p < 0.01.

Data presented in this study were obtained from VIP-eGFP mice [MMRRC strain#31009, STOCK Tg(Vip-EGFP)37Gsat, CD1 genetic background, University of California, Davis, CA, United States]. In this mouse line, virtually all interneurons that express VIP endogenously were confirmed to also express eGFP using a rabbit anti-VIP primary antibody (catalog #20077, 1:400; Immunostar; see Figure 1A,B in Tyan et al., 2014 and Supplementary Figure 2G in Francavilla et al., 2018). The animals were separated into two experimental groups: young (P90-150) and old (P410–680). The age range of old group was determined by a low life duration of VIP-eGFP mice, with most animals being lost by P520 (Figure 1A). For all experiments both males and females were used (young: *n* = 46 vs. old: *n* = 34). All experiments were conducted in accordance with the Animal Protection Committee of Université Laval and the Canadian Council on Animal Care.

### Behavior Assays

General exploratory activity in the open field was assessed using the automated VersaMax Animal Activity Monitoring System (AccuScan Instruments, Columbus, OH). The open field was a square arena (20 × 20 × 30.5 cm) made of plexiglass equipped with photocell beams for automatic detection of activity. On day 1, mice were placed in the open field (Figure 1C, left) and left to freely explore for a 10 min test session. The number of vertical beam breaks was taken as a measure of vertical activity. The stereotypic activity was detected automatically using the VersaMax software (Omnitech Electronics Inc, OH, United States). Test chambers were cleaned with 70% ethanol between subjects.

The object-place task was performed in the same arena used for open field. On day 2, during the Sample phase (Figure 1C, middle), a single mouse was placed in the arena containing two different sample objects (A + B; 4 × 4 cm each) for 10 min, after which it was returned to its home cage for 24 h retention interval. On day 3, during the Test phase (Figure 1C, right), mice re-entered the arena with two objects, one was identical to the Sample phase and the other was novel (C + A; 4 × 4 cm each). To reinforce the object novelty aspect of this test, we altered the spatial location of the old object explored by the mouse. The results are expressed as Recognition Index (RI, %), defined as the ratio of the time spent with new object divided by the total exploration time for novel and familiar objects [RI = (*T*_C_)/(*T*_C_ + *T*_A_)]. An exploration time was also calculated for the time spent with the two objects in the Test phase (ET_T_ = *T*_C_ + *T*_A_).

For Morris water maze (MWM) test, a white, circular, polypropylene pool (158 cm in diameter, 60 cm height) that was filled with water (21°) made opaque by the addition of powdered milk was used. A clear Plexiglas, adjustable platform (35 cm height, 14 cm circumference), was submerged 2.0 cm below the water surface or elevated 0.5 cm above the water level. The proximal cues comprising a black cardboard in the shape of a star, a triangle, a cylinder, or a cube (10 × 7 × 2.5 cm) hung on the pool walls above the water level at one of four possible starting points (e.g., north, south, east, west) (Figure 1G). Mice (*n* = 10, young; *n* = 8 old) received five trials in the MWM during 4 subsequent days. In all trials, an individual mouse was placed into the water, facing the outer edge of the pool, at one of four possible starting points (e.g., north, south, east, west). The starting location for each trial was determined randomly. A trial was terminated, and the latency was recorded when the animal reached the platform and remained on it for 10 s. If the animal did not reach the platform within 60 s, the trial was terminated by placing the animal on the platform for 10 s. After the trial, animals were transferred to a dry holding cage where they remained for 60 s until the next trial. During testing, the submerged platform remained stationary in one quadrant of the maze, and the latency to find the platform as well as the distance traveled within the target quadrant, the time spent within the target area and the number of entries to this area were recorded using the Any-Maze software (Stoelting, IL, United States). The distance in target area was then expressed as a fraction of the total distance (in %), while the time in target area was expressed as a fraction of the total time of exploration.

For the anxiety test, the elevated plus maze (EPM) was used (Figure 1I). The maze was made of beige plexiglas and consisted of four arms (30 × 5 cm) elevated 40 cm above floor level. Two of the arms contained 15 cm high walls and ceiling (enclosed arms) and the other two none (open arms). Each mouse was placed in the middle section facing a close arm and left to explore the maze for 10 min. After each trial, the floor of the maze was wiped clean with 70% ethanol and dried. The total distance traveled, open arm entries and duration of stay within open arm zones were recorded using the Any-Maze software.

### Slice Preparation and Patch-Clamp Recordings

Coronal hippocampal slices (thickness, 300 μm) were prepared from VIP-eGFP mice of either sex. Briefly, animals were anesthetized deeply with ketamine-xylazine mixture (10/100 mg/mL), transcardially perfused with 25 mL of ice-cold cutting solution (containing the following in mM: 250 sucrose, 2 KCl, 1.25 NaH_2_PO_4_, 26 NaHCO_3_, 7 MgSO_4_, 0.5 CaCl_2_, and 10 glucose, pH 7.4, 330–340 mOsm/L) and decapitated. Slices were cut in the cutting solution using a vibratome (VT1000S; Leica Microsystems or Microm; Fisher Scientific), and transferred to a heated (37.5°C) oxygenated recovery solution containing the following (in mM): 124 NaCl, 2.5 KCl, 1.25 NaH_2_PO_4_, 26 NaHCO_3_, 3 MgSO_4_, 1 CaCl_2_, and 10 glucose; pH 7.4; 300 mOsm/L, and allowed to recover for 45 min. During experiments, slices were continuously perfused (2 mL/min) with standard artificial cerebrospinal fluid (ACSF) containing the following (in mM): 124 NaCl, 2.5 KCl, 1.25 NaH_2_PO_4_, 26 NaHCO_3_, 2 MgSO_4_, 2CaCl_2_, and 10 glucose, pH 7.4 saturated with 95% O_2_ and 5% CO_2_ at near physiological temperature (30–33°C). VIP-positive interneurons (VIP+) located in CA1 stratum radiatum (RAD) and pyramidale (PYR) were visually identified as eGFP-expressing cells under an epifluorescence microscope with blue light (filter set: 450–490 nm). All electrophysiological recordings were carried out using a 40x water-immersion objective. Two-photon images of eGFP-expressing interneurons were acquired in the same layers of acute slices obtained from VIP-eGFP mice, using a two-photon microscope (TCS SP5; Leica Microsystems) based on a Ti-Sapphire laser tuned to 900 nm (Figure 1B). Images were acquired with a 25x water-immersion objective (NA 0.95). A Flaming/Brown micropipette puller (Sutter Instrument Co.) was used to make borosilicate glass capillaries (3.5–6 MΩ). Whole-cell patch-clamp recordings from VIP+ interneurons were performed in voltage or current-clamp mode. Pipettes filled with K^+^-based solution were used for current-clamp recordings from VIP+ interneurons located in RAD and PYR (in mM): 130 KMeSO_4_, 2 MgCl_2_, 10 di-Na-phosphocreatine, 10 HEPES, 4 ATP-Tris, 0.4 GTP-Tris, and 0.3% biocytin (Sigma), pH 7.2–7.3, 280–290 mOsm/L. Passive and active membrane properties were analyzed in current clamp mode: active membrane properties were recorded by subjecting cells to multiple current step injections of varying amplitudes (–200 to 200 pA). Passive membrane properties (resting membrane potential, input resistance, and membrane capacitance) were obtained immediately after membrane rupture. Series resistance (in voltage-clamp) or bridge balance (in current-clump) were monitored throughout the experiment. In current-clamp experiments, interneurons were held at –70 mV by injecting current (–10 to –30 pA), if necessary. Voltage-clamp recordings were performed to analyze the excitatory and inhibitory drives received by VIP+ cells. Cells that had a resting membrane potential more positive than –45 mV, or showed an increase in holding current (>–30 pA) or changes in series resistance or bridge balance (>15%) during recording were discarded. For recordings of spontaneous excitatory postsynaptic currents (sEPSCs), whole-cell patch-clamp recordings were performed in voltage-clamp at –70 mV. Spontaneous inhibitory postsynaptic currents (sIPSCs) and miniature inhibitory postsynaptic currents (mIPSCs) were recorded in voltage-clamp at 0 mV in the presence of tetrodotoxin (TTX; 1 μM; Alomone Labs). For all voltage-clamp recordings we used an intracellular Cs^+^-based solution containing (in mM): 130 CsMeSO_4_, 5 CsCl, 2 MgCl_2_, 10 phosphocreatine, 10 HEPES, 0.5 EGTA, 4 ATP-TRIS, 0.4 GTP-TRIS, 0.3% biocytin, 2 QX-314 (pH 7.2–7.3; 280–290 mOsm/L). For the isolation of voltage-dependent delayed rectifier potassium 3.1 (Kv3.1) currents (Hernandez-Pineda et al., 1999), voltage-clamp recordings in whole-cell configuration with holding membrane potential at –40 mV were performed. In these recordings, extracellular solution was supplemented with 1 μM TTX and Cadmium Chloride (CdCl_2_, 100 μM; Sigma) to block voltage-dependent sodium and calcium currents, respectively. Depolarizing pulses were applied from –40 to +30 mV with an increment of +10 mV and recordings were performed both in the presence and in the absence of tetraethylammonium (TEA, 1 mM; Sigma) applied to the extracellular solution. Data acquisition (filtered at 2–3 kHz and digitized at 10 kHz; Digidata 1440, Molecular Devices, CA, United States) was performed using the Multiclamp 700B amplifier and the Clampex 10.5 software (Molecular Devices).

### Electrophysiological Data Analysis

Analysis of electrophysiological recordings was performed using Clampfit 10.6 (Molecular Devices). For the analysis of the AP properties, the first AP appearing at the rheobase current pulse within a 50 ms time window was analyzed. The AP amplitude was measured from the AP threshold to the peak. The AP half-width was measured at the voltage level of the half of AP amplitude. The AP rise time was detected between the AP threshold and the maximal AP amplitude, while the AP fall time between the maximal AP amplitude and the AP end. The hyperpolarization-activated cation current (Ih)-associated voltage rectification was determined as the amplitude of the membrane potential sag from the peak hyperpolarized level to the level at the end of the hyperpolarizing step when the cell was hyperpolarized to –100 mV. The membrane time constant (τ) was measured offline using an exponential fit of voltage response to a hyperpolarizing current step of –40 to –50 pA. The firing pattern in I-S3 cells was assessed at 2x rheobase current. The maximum firing frequency was obtained from the inter-spike interval between the first two APs evoked by a current pulse of +140–150 pA. The number of APs was assessed at the current pulse of +140–150 pA.

For the analysis of spontaneous synaptic currents, a minimum of 100 events (for EPSCs) and 200 events (for IPSCs) were sampled per cell over a 10 min period using an automated template search algorithm in Clampfit. All events were counted for frequency analysis. The inhibitory (Gi) or excitatory (Ge) synaptic conductance (Figure 6E) was calculated as G = I/(V_hold_ – V_rev_). Charge transfer (Figure 7D) was calculated by integrating the area under the PSC waveform. The mean PSC synaptic current (Figures 6F, 7E) was calculated as the charge transfer of the averaged PSC multiplied by mean PSC frequency (Park et al., 2006; Potapenko et al., 2011).

For the isolation of Kv3.1 currents (TEA-sensitive component), the digital subtraction of the TEA-insensitive component from the total current obtained without TEA application was performed (Hernandez-Pineda et al., 1999). For computing the conductance, a K^+^ reversal potential of –95 mV was assumed (Lien et al., 2002). For fitting activation curves, a non-linear least squares algorithm (Lien et al., 2002) was used to fit the following sigmoid: G/Gmax(V) = 1/(1 + exp(−(V − Vhalf)/k)).

### Anatomical Reconstruction and Immunohistochemistry

**FIGURE 2:**
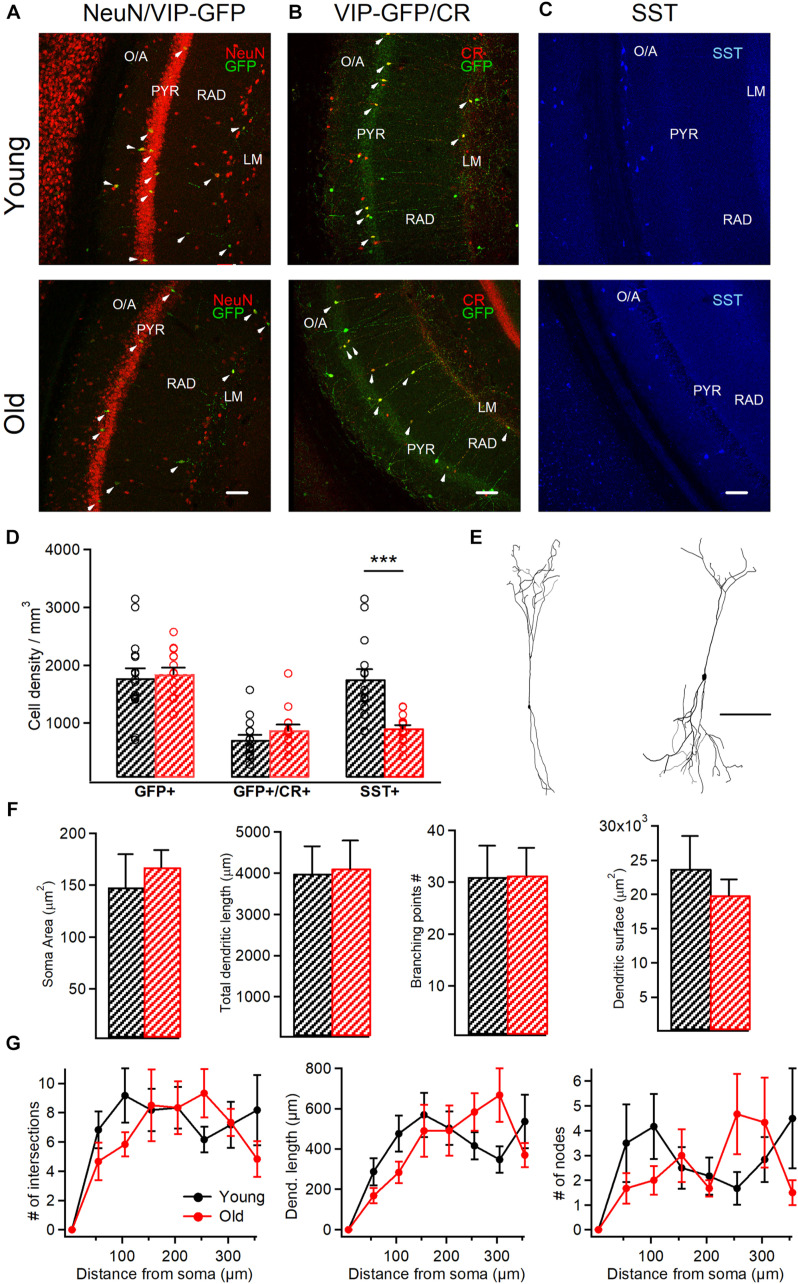
VIP+/CR+ interneurons exhibit no changes in morphological properties in aged mice. **(A)** Representative confocal images of the hippocampal CA1 area of VIP-eGFP mice showing immunoreactivity for eGFP and NeuN **(A)**, eGFP and CR **(B)** and SST **(C)** in young and old mice. White arrowheads point to cells showing the colocalization of eGFP and NeuN **(A)** or eGFP and CR **(B)**. Scale bars: 100 μm. **(D)** Summary data showing no change in the cell density for VIP-eGFP and VIP-eGFP+/CR+ interneurons (*p* > 0.05, unpaired *t*-test) but a significant decrease in SST+ interneurons (*p* < 0.001, ***p < 0.01, unpaired *t*-test) between the two age groups. **(E)** Anatomical reconstructions of I-S3 cells filled with biocytin during whole-cell patch-clamp recordings. Scale bar: 100 μm. **(F)** Summary data showing no differences in somatic and dendritic parameters between the two age groups (*p* > 0.05 for all comparisons, unpaired *t*-test/ Mann-Whitney test). **(G)** Sholl analysis of I-S3 dendritic branch patterns revealed no differences in the number of dendritic intersections (left), nodes (right), or dendritic length (middle) (*p* > 0.05 for all comparisons, unpaired *t-*test/ Mann-Whitney test).

For *post hoc* reconstruction, neurons were filled with biocytin (Sigma) during whole-cell recordings. Slices with recorded cells were fixed overnight with 4% paraformaldehyde (PFA) at 4°C. To reveal biocytin, the slices were permeabilized with 0.3% Triton X-100 and incubated at 4°C with streptavidin conjugated Alexa-488 (1:1,000) in Trizma-buffer (TB). Z-stacks of biocytin-filled cells were acquired with a 1 μm step using a 20x oil-immersion objective and Leica SP5 confocal microscope. Confocal stacks were merged for detailed reconstruction in Neurolucida 8.26.2 (MBF Bioscience). The I-S3 cell phenotype was confirmed by the presence of axon in the O/A (Acsády et al., 1996b; Tyan et al., 2014). The quantitative analysis of soma size and dendritic morphology was performed following 3D reconstructions of I-S3 cells in Neurolucida. Sholl analysis (Figure 2G) was performed in radial coordinates, using a 50 μm step size from *r* = 0, with the origin centered on the cell soma, and counting the number of compartments crossing a given radius.

All immunohistochemical tests were performed on free-floating sections (50–70 μm thick) obtained with Leica VT1000S or PELCO EasySlicer vibratomes. VIP-eGFP mice were deeply anaesthetized with ketamine–xylazine mixture (ketamine: 100mg/kg, xylazine: 10mg/kg) and perfused intracardially with 4% PFA, and the brains were sectioned. Coronal sections (AP; –2.0–2.8; *n* = 20 sections/age group, 6–7 sections/animal from 3 different mice) were permeabilized with 0.25% Triton X-100 in PBS and incubated overnight at 4°C with primary antibodies followed by the secondary antibodies. The following primary antibodies were used in this study: goat anti-CR (1:1,000; Santa Cruz, #sc-11644), chicken anti-GFP (1:1,000; Aves, #GFP-1020), mouse anti-NeuN (1:500; Millipore, #MAB377), and rat anti-SST (1:500; Millipore, #MAB354). In control immunohistochemical tests, the primary antibodies were omitted, and sections were incubated with the secondary antibodies only. Confocal images of the hippocampal CA1 area were acquired sequentially using a 20x oil-immersion objective (0.8 NA) and Leica TCS SP5 imaging system coupled with a 488 nm argon, a 543 nm HeNe and a 633 nm HeNe lasers. The imaging parameters were kept constant across all sections/animals. Cells were considered immunopositive when the corresponding fluorescence intensity was at least twice the background fluorescence. For representation only, the overall brightness and contrast of images were adjusted manually. Portions of images were not modified separately in any way.

Cell counting was performed blind to the experimental conditions within the CA1 area using stereological analysis. The cells were first counted in different sections (*n* = 6–7) originating from the same animal, and the data was then compared between different animals (*n* = 3 animals/age group) and pooled together for young vs. old groups. Cell density was estimated within regions of interest (500 × 700 μm) selected randomly in the middle of the CA1.

### Multi-Compartment Models of I-S3 Cells

For multi-compartment modeling we used previously developed I-S3 cell models (Guet-McCreight et al., 2016), and simulations were done using the NEURON software environment (Carnevale and Hines, 2006). We included two model variants, which either had A-type K^+^ current in the proximal dendrites (SDprox1) or had A-type K^+^ current restricted to the soma (SDprox2). Because it remains unclear what the morphological distributions of A-type K^+^ currents are in I-S3 cells, we used both model variants in this study. Both models possess only a minimal complement of ion channel mechanisms to capture the basic spiking activity of I-S3 cells (Guet-McCreight et al., 2016). These include transient Na^+^ current, persistent Na^+^ current, A-type K^+^ current, fast delayed rectifier K^+^ current, and random Gaussian noise current to capture subthreshold fluctuations and irregular firing observed in I-S3 cell recordings. To probe the possible channel currents that might be modulated given observed changes in I-S3 cell active properties during aging, we looked at a variety of changes in these ion channel conductances in the I-S3 cell models, and selected the conductance change that was able to best capture the changes that are seen experimentally. Note that these simulations were conducted in a systematic way, where conductances were either reduced from 100 to 0% of their initial values or increased from 100 to 200% of their initial values. As such, there were no attempts to fit the aging electrophysiology data or perform parameter searches; we were simply looking at the effects of upscaling or downscaling the ion channel conductances in a way that was illustrative. Simulated voltage traces were analyzed in Python 3 using the eFEL module. Specifically, we used this module to obtain measurements for the AP amplitude, half-width, rise rate, and fall rate. The AP rise and fall rates can both be described as the ratio of the voltage differences (rise/fall voltages) and the time differences (rise/fall times) as calculated using the AP begin, end, and peak indices.

### Statistics

For statistical analysis, distributions of data were first tested for normality with a Shapiro–Wilcoxon test. If data were normally distributed, standard parametric statistics were used: unpaired two-tailed *t*-tests for comparisons of two groups and one-way or repeated-measures ANOVA for comparisons of multiple groups followed by Tukey, Kruskal–Wallis or Chi^2^ *post hoc* tests. If data were not normally distributed, non-parametric Mann–Whitney test was instead used for comparisons of two groups. The Kolmogorov-Smirnov (K-S) test was used for comparison of spontaneous synaptic events. All statistical analysis was conducted in Sigma Plot 11.0, Clampfit 10.6 and Igor Pro. The “*p*” values < 0.05 were considered significant. Error bars correspond to standard error of the mean (SEM). Error bars for potassium conductances and kinetics correspond to standard deviations, and shaded areas correspond to SEM.

## Results

### VIP-eGFP Mice Display Age-Dependent Behavioral Deficits

**FIGURE 3:**
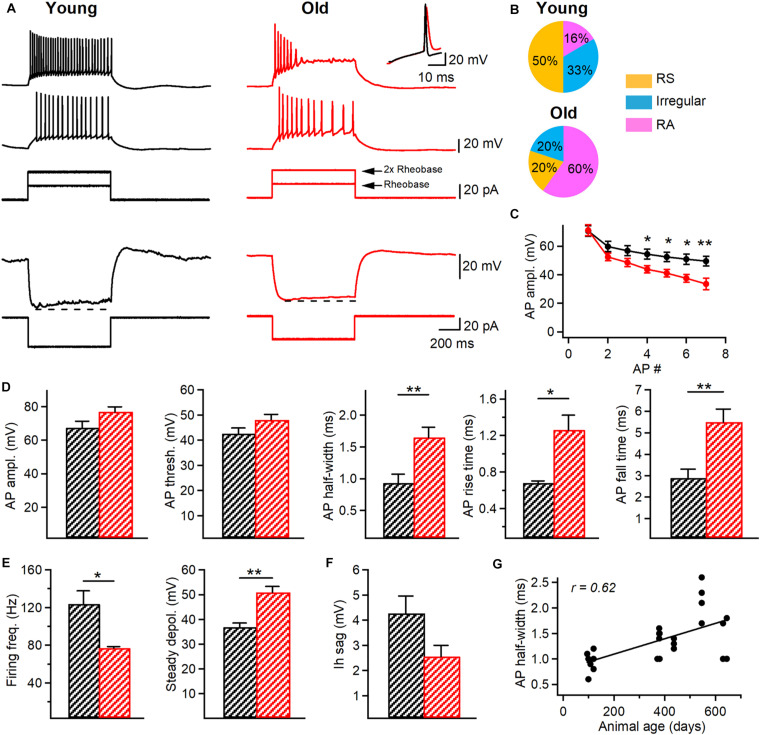
Changes in the firing properties of I-S3 cells of aged mice. **(A)** Top, representative voltage responses (top) indicating the firing pattern of I-S3 cells in young (black) and old (red) mice in response to depolarizing (+20 pA and +40 pA) current injections corresponding to rheobase and 2x rheobase current, respectively. Inset on the top right corresponds to the superimposed traces showing the longer AP duration in old mice. Bottom, representative voltage responses of I-S3 cells in the two age groups to a hyperpolarizing current injection used to assess the Ih-associated voltage rectification (dashed line). **(B)** Pie charts illustrating the percentage of the regularly spiking (RS), irregular spiking (Irregular) and rapidly adapting (RA) firing patterns in I-S3 cells from the two age groups. **(C)** Summary plot (mean ± SEM) showing changes in the AP amplitude during the train of APs in I-S3 cells (*p < 0.05, **p < 0.01, unpaired *t*-test). **(D)** Summary data showing no changes in the AP amplitude and AP threshold between young and old mice (*p* > 0.05, unpaired *t-*test) but a significant increase in the AP half-width (*p* < 0.01, unpaired *t*-test), rise time (*p* < 0.05, unpaired *t*-test) and fall time (*p* < 0.01, unpaired *t*-test). **(E)** Summary data for two groups of animals showing a significant decrease in the maximal firing frequency (*p* < 0.05, unpaired *t*-test) in parallel with an increase in the steady state depolarization (*p* < 0.01, unpaired *t-*test). **(F)** Summary data for two groups of animals showing no changes in the Ih sag (*p* < 0.05, unpaired *t*-test). **(G)** Positive correlation between the AP half-width and the animal age (*R* = 0.62, *p* < 0.01, Spearman correlation).

To examine the impact of aging on the morphological and electrophysiological properties of VIP+/CR+ IS3 cells, VIP-eGFP mice were used. As demonstrated previously (Tyan et al., 2014), in this mouse model, all interneurons immunoreactive for VIP express eGFP, and VIP+ cells of different subtypes, including bipolar/bitufted IS3 cells with soma located within the CA1 PYR and RAD (Figure 1B), can be targeted for patch-clamp recordings. To define the age of experimental animals to be included in the study, we first examined the longevity of VIP-eGFP mice, which have a CD1 genetic background. We found that, differently from C57BL/6 mice (Goodrick, 1975), VIP-eGFP mice had shorter life duration (P365–660), with most animals dying by P500 (Figure 1A). Accordingly, for all experiments on old mice we choose the age range of P420–P680 (the oldest possible animals in our colony) (*n* = 34 mice). The young group included animals from P90 to P160 (*n* = 46 mice) (Figure 1A). In some experiments (Figures 1E, 3G), an additional age group (P350–410; *n* = 29 mice) was included for age-dependent correlation analyses.

The age-dependent decline in spatial memory has been reported in different lines of mice previously (Fordyce and Wehner, 1993; Bach et al., 1999). To examine whether this is the case for VIP-eGFP mice, we compared the performance of young vs. old animals in the object-place test (OPT) and the Morris water maze (MWM) (Figures 1C–H). We first determined whether aging is associated with a decline in locomotor performance by analyzing the activity of VIP-eGFP mice in the open field maze (OFM) (Figures 1C,D). Old mice showed a smaller total distance traveled (young: *n* = 9 vs. old: *n* = 8, *p* < 0.05, *t*-test, Figure 1D, left), with no change in rearing events (young: *n* = 9 vs. old: *n* = 8, *p* > 0.05, *t*-test, Figure 1D, middle) and stereotypy counts (young: *n* = 9 vs. old: *n* = 8, *p* > 0.05, *t*-test, Figure 1D, right). Overall, these data indicate a decreased locomotor activity in old VIP-eGFP mice. On day 2, mice were tested in the OPT. The data showed that, during the Sample phase (Figure 1C, middle), mice of both age groups were spending similar amount of time exploring two different sample objects (A and B) as indicated by their RI (young: *n* = 9 vs. old: *n* = 8, *p* > 0.05, *t*-test, Figure 1F, left). However, 24 h later, once the mice were re-introduced in the same maze containing a new object (C) and the sample object (A) positioned in a different spatial location (Figure 1C, right), a significant difference was observed in the RI for the novel object recognition betwee young and old mice (*p* < 0.05, *t-*test, Figure 1F, middle), pointing to a decline in the novelty recognition with age. Indeed, when additional age group of animals (P350–410; *n* = 29) was included in this analysis, the decline in the novel object recognition RI correlated well with the mouse age (*R* = 0.41, *p* < 0.001, Spearman correlation, Figure 1E). Importantly, the total exploration time during the OPT was not significantly different between the two age groups (*p* > 0.05, *t-*test, Figure 1F, right), indicating that the lower RI in the aged group reflects poorer memory performance and not decreased exploratory behaviors. To investigate the potential spatial memory deficits in old mice, we next performed the MWM test (young: *n* = 10 mice, old: *n* = 8 mice; Figures 1G,H). The data showed that old VIP-eGFP mice showed a significantly longer escape latency to find a fixed hidden platform (*p* < 0.01, one-way ANOVA; Figure 1G, right). Also, on the final day of training, old mice demonstrated a lower distance traveled (*p* < 0.01, *t-*test) and time spent (*p* < 0.01, *t-*test) in the target quadrant with fewer entries into the target zone (*p* < 0.05, *t-*test) (Figure 1H). Taken together, these data indicate that old VIP-eGFP mice exhibit significant impairments in the hippocampus-dependent memory.

Together with memory performance deficits, aging has also been associated with an increase in the anxiety-like behavior (Gorina et al., 2017). To test whether this is the case in aged VIP-eGFP mice, we performed the EPM test (young: *n* = 8 mice, old: *n* = 7 mice; Figures 1I,J). Our data showed that old VIP-eGFP mice spent much less time in the open arms (*p* < 0.001, *t-*test; Figures 1I,J, center) with less entries into open arms (*p* < 0.001, *t-*test; Figures 1I,J, right), indicative of increased anxiety although decreased locomotion could also account for these changes. Thus, similar to other mouse strains, in VIP-eGFP mice, aging is associated with decreased locomotion, memory deficits, and anxiety-like behavior.

### I-S3 Cells Survive During Aging and Preserve Their Morphology

To investigate whether aging has an impact on the survival of CA1 VIP+ interneurons, we performed immunohistochemistry on hippocampal slices from young *vs.* old VIP-eGFP mice (young: *n* = 8 mice; old: *n* = 5 mice), using antibodies against NeuN to label all neurons and GFP to label all VIP-expressing interneurons (Tyan et al., 2014; Francavilla et al., 2018). We found that VIP+ interneurons survive during aging as no change in their cell density was measured in the CA1 area (*p* > 0.05, *t*-test; Figures 2A,D). Furthermore, our data showed no difference in the VIP+ population co-expressing CR and VIP and corresponding to I-S3 cells (*p* > 0.05, *t*-test; Figures 2B,D), indicating that I-S3 interneurons in the hippocampal CA1 area also survive during animal aging. These data contrasted with the density of SST+ GABAergic interneurons located within CA1 O/A (Figures 2C,D), which was significantly decreased in old VIP-eGFP mice (*p* < 0.001, *t*-test).

As aging is often associated with changes in morphological properties of neurons, such as reduced dendritic branching (Scheibel, 1979; Hanks and Flood, 1991), we next explored the age-dependent structural changes in the anatomically confirmed I-S3 interneurons that were filled with biocytin during patch-clamp recordings and reconstructed in Neurolucida (young: *n* = 4 cells vs. old: *n* = 6 cells; Figure 2E). Our data showed that aging had no impact on I-S3 cell morphology. In particular, no difference was found in such morphological parameters as soma area (*p* > 0.05, *t*-test), total dendritic length (*p* > 0.05, *t*-test), number of branching points (*p* > 0.05, Mann-Whitney test) and area occupied by dendrites (*p* > 0.05, *t*-test) (Figure 2F). Furthermore, dendritic Sholl analysis revealed no differences in the number of dendritic intersections (*p* > 0.05, one-way ANOVA; Figure 2G, left), dendritic length (*p* > 0.05, one-way ANOVA; Figure 2G, middle) or number of dendritic nodes (*p* > 0.05, one-way ANOVA; Figure 2G, right) at different distances from the soma. Together, these data indicate that I-S3 cells survive at later stages of the animal’s life and preserve their morphology.

### Aging Modifies the Physiological Properties of I-S3 Cells

As cognitive deficits during aging can be associated with changes in electrophysiological properties of neurons (Rizzo et al., 2014), we performed targeted patch-clamp recordings from I-S3 cells in slices obtained from young (*n* = 9 cells) vs. old (*n* = 11 cells/) VIP-eGFP mice, and analyzed the active and passive membrane properties of these cells (Figure 3). We found no changes in the resting membrane potential (V_m_ young: –59.8 ± 1.7 mV; V_m_ old: –60.6 ± 1.7 mV; *p* > 0.05, *t*-test), input resistance (R_in_ young: 619.7 ± 78 MΩ; R_in_ old: 486 ± 61 MΩ; *p* > 0.05, *t*-test), membrane capacitance (C_m_ young: 34.1 ± 3.3 pF; C_m_ old: 27.3 ± 2.3 pF; *p* > 0.05, *t*-test) or membrane time constant (τ_m_ young: 31.1 ± 2.0 ms; τ_m_ old: 31.4 ± 3.8 ms; *p* > 0.05, *t*-test) in I-S3 cells of old mice. However, analysis of active membrane properties revealed significant changes in I-S3 firing and AP properties (Figure 3A). First, while in young mice I-S3 cells exhibited a regularly spiking (RS), an irregularly spiking or a rapidly adapting (RA) firing pattern (Figure 3A, left and Figure 3B, top), the RA firing pattern was seen more often in old animals (Figure 3A, right and Figure 3B, bottom), revealing a stronger firing adaptation in I-S3 cells in aged mice. Moreover, there was a significant decrease in the amplitude of spikes during the train in old animals (*p* < 0.05, *t-*test; Figure 3C). Furthermore, the level of the steady-state depolarization was significantly higher in old animals (young: 36.8 ± 1.7 mV; old: 50.9 ± 2.4 mV; *p* < 0.01, *t-*test; Figure 3E) in parallel with a significant decrease in the maximal firing frequency (young: 123.7 ± 13.9 Hz; old: 76.9 ± 1.6 Hz; *p* < 0.05, *t*-test; Figure 3E). The detailed analysis of the first AP in the train recorded at the rheobase current showed that while aging had no impact on the AP amplitude (young: 67.4 ± 3.9 mV; old: 76.7 ± 2.9 mV; *p* > 0.05, *t-*test) and threshold (young: –42.6 ± 2.2 mV; old: –48.1 ± 2.1 mV; *p* > 0.05, *t*-test), the AP half-width (young: 0.9 ± 0.1 ms; old: 1.6 ± 0.2 ms; *p* < 0.01, Mann-Whitney test), rise time (young: 0.68 ± 0.02 ms; old: 1.26 ± 0.16 ms; *p* < 0.05, Mann-Whitney test) and fall time (young: 2.9 ± 0.4 ms; old: 5.5 ± 0.6 ms; *p* < 0.01, *t*-test) were significantly increased (Figure 3D). Importantly, when an additional set of data from I-S3 cells of younger mice (P370–380, *n* = 6 cells) was included in this analysis, there was a positive correlation between the AP half-width and the animal’s age (*R* = 0.62, *p* < 0.05, Spearman correlation, Figure 3G), in line with the age-dependent progress of changes in the AP kinetics. We found no changes in the hyperpolarization-activated membrane conductances, such as I_h_ current (V_m_ sag young: 4.3 ± 0.7 mV; V_m_ sag old: 3.2 ± 0.9 mV; *p* > 0.05, *t-*test; Figure 3F), pointing to specific age-dependent changes in voltage-gated channels involved in the generation of APs in I-S3 cells.

### Modeling Provides Insights Into the Age-Related Changes Occurring in I-S3 Cells

To probe the possible changes in intrinsic properties associated with age-related modifications of active properties of I-S3 cells, we used two model variants of the previously developed multi-compartment computational models of I-S3 cells (Guet-McCreight et al., 2016): a variant possessing dendritic A-type potassium channels (SDprox1) and one lacking dendritic A-type potassium channels (SDprox2).

**FIGURE 4:**
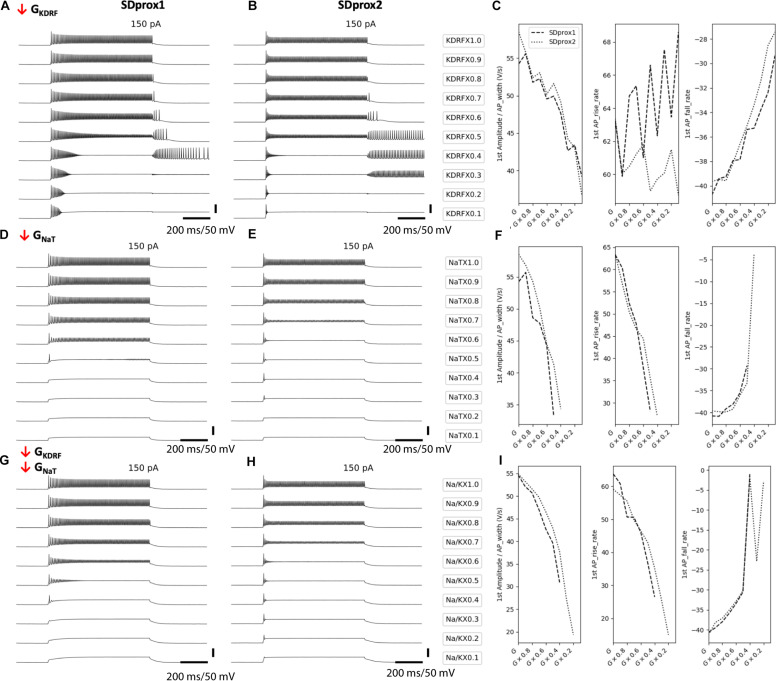
Downscaling fast delayed rectifier potassium and transient sodium channels in I-S3 cell models mimics the cell firing properties in aged animals. **(A,B)** Simulated voltage recordings of I-S3 cell spiking using models SDprox1 **(A,D,G)** and SDprox2 **(B,E,H)**, respectively. From top to bottom, fast delayed rectifier potassium conductance **(A,B)**, transient sodium conductance **(D,E)**, and both **(G,H)** are downscaled from 100% of its initial value to 10%. **(C,F,I)** Changes in ratio of the first AP amplitude over half-width (V/s) (left panels), first AP rise rate (middle panel), and first AP fall rate (right panel) as conductances are downscaled. Note that the AP rise and fall rates are computed as the ratio of the voltage difference over time difference (V/s) of the rise and fall portions of the AP, respectively. For all three measurements, values closer to zero (i.e., slower rates) correspond with slower durations.

In upscaling and downscaling several intrinsic ion channel conductances we observed that the age-related changes seen experimentally were best captured when fast delayed rectifier potassium conductances were reduced (Figures 4A–C), when transient sodium conductances were reduced (Figures 4D–F), and when both were reduced proportionally (Figures 4G–I). Generally, reducing these conductances allowed the models to enter the depolarization block states more easily, most likely due to a change in the balances between sodium to potassium currents (Bianchi et al., 2012; Tucker et al., 2012). More specifically, reducing these channel conductances in the models led to increases in the first spike half-width rates, as scaled by spike amplitude (Figures 4C,F,I, left panels). We scaled the spike half-widths by spike amplitude to disambiguate spike duration measurements from effects due to changes in spike amplitude. Correspondingly, we also measured the spike rise rate (V/s; Figures 4C,F,I, middle panels) and spike fall rates (V/s; Figures 4C,F,I, right panels). We found that reducing fast delayed rectifier potassium conductance leads to slower spike fall rates (Figure 4C, right panel), but does have a large impact on spike rise rates (Figure 4C, middle panel). On the other hand, reducing transient sodium conductance results in slower spike rise rates (Figure 4F, middle panel), with some impact at slowing spike fall rate as well (Figure 4F, right panel). Reducing both conductances had an effect of slowing both the spike rise and fall rates (Figure 4I). Kinetically, the fast delayed rectifier potassium channel model used in the I-S3 cell models most likely corresponds with currents generated by the Kv3.1 potassium channel subunits (Guet-McCreight et al., 2016) and the transient sodium channel could correspond with sodium channel subunits Nav1.2, Nav1.3, and beta-2.

Since these models possess a minimal complement of ion channel mechanisms (see Methods) and I-S3 cell ion channel conductances and kinetics remain largely uncharacterized, we did not attempt to fit the aging data more closely. Therefore, while these simulations suggest that changes in fast delayed rectifier potassium and transient sodium conductances contribute to the aging-related changes seen in electrophysiological recordings from I-S3 cells, they do not exclude other intrinsic mechanisms.

### The TEA-Sensitive and Insensitive K^+^ Currents Remain Unaltered in I-S3 Cells of Old Mice

**FIGURE 5:**
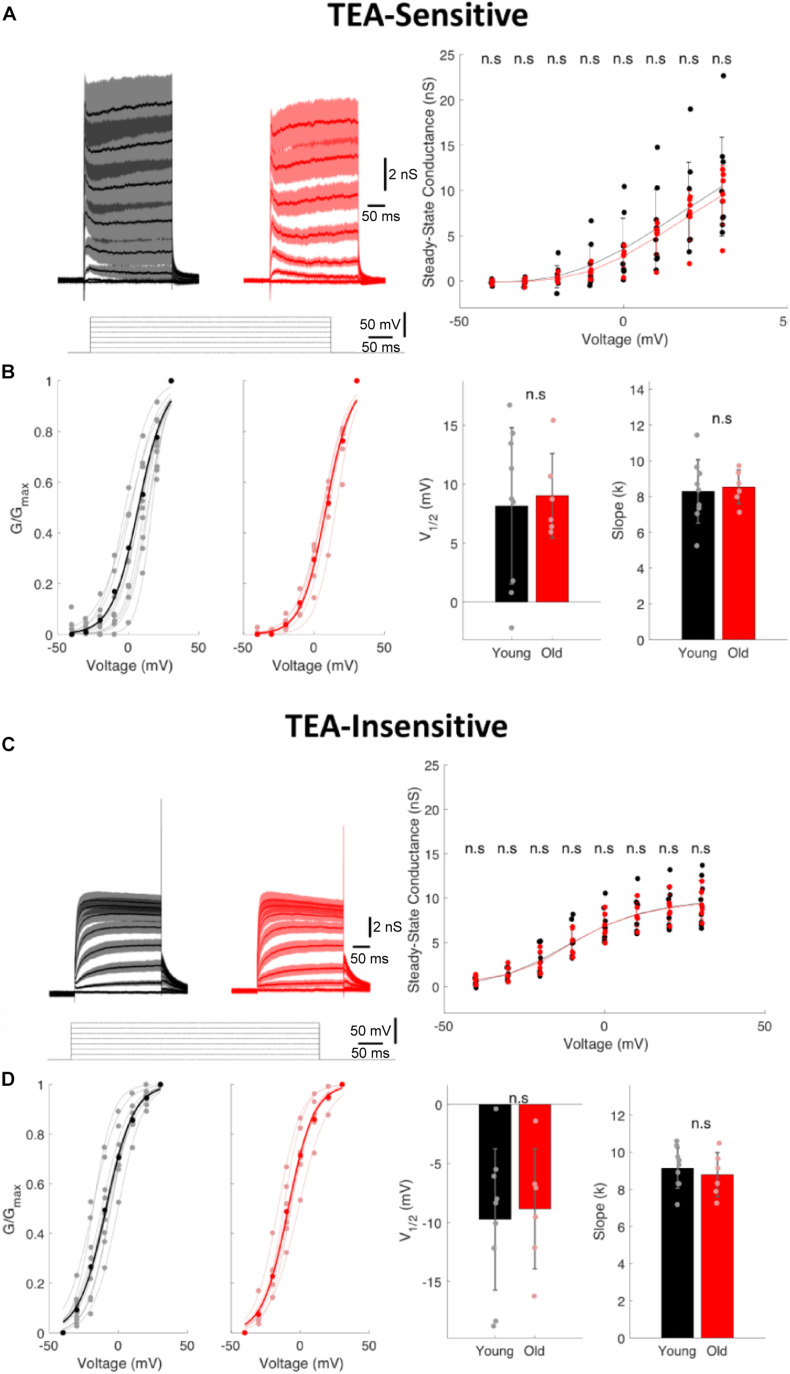
The TEA-sensitive and TEA-insensitive potassium currents remain unaltered in I-S3 cells. **(A)** Left, currents obtained from an I-S3 cell held at –40 mV and subjected to depolarizing pulses from –40 to +30 mV in 10 mV increments after application of 1 mM TEA in young (black) and old (red) mice. Traces show the TEA-sensitive component obtained by digital subtraction of the traces recorded in the presence of TEA (**C**, left; TEA-insensitive currents) from those in control (total currents). Right, the steady-state conductance of the TEA-sensitive component as a function of voltage in young and old mice showing no changes in the TEA-sensitive currents (*p* > 0.05, unpaired *t*-test; error bars show standard deviation). **(B)** Left, plots of relative conductance (G/Gmax) of the TEA-sensitive current in I-S3 cells of young and old mice with a Boltzmann fit function. Right, summary data showing no significant change in V_1/2_ and k of the TEA-sensitive currents (*p* > 0.05, unpaired *t*-test; error bars show standard deviation). **(C)** Left, current traces recorded in the presence of TEA during the depolarizing pulses from –40 to +30 mV in 10 mV increments and corresponding to the TEA-insensitive component in young (black) and old (red) mice. Right, the steady-state conductance of the TEA-insensitive component as a function of voltage in young and old mice showing no difference in this component between the two age groups (*p* > 0.05, unpaired *t-*test; error bars show standard deviation). **(D)** Left, plots of relative conductance (G/Gmax) of the TEA-insensitive current in young and old mice fit to a Boltzmann function. Right, summary data showing no significant change in V_1/2_ and k of the TEA-insensitive currents (*p* > 0.05, unpaired *t*-test; error bars show standard deviation).

To test the model predictions that the down-regulation of Kv3.1 channels can be responsible for age-related increases in the steady-state depolarization and AP duration in I-S3 cells, we performed voltage-clamp recordings in whole-cell configuration with holding membrane potential at –40 mV to facilitate the isolation of Kv3.1 currents from I-S3 cells in young and old VIP-eGFP mice (young: *n* = 9 cells; old: *n* = 6 cells). To isolate the TEA-sensitive Kv3.1-mediated current (Figure 5A, left), we subtracted the TEA-insensitive component (Figure 5C, left) from the total current recorded before application of TEA. TEA-sensitive and TEA-insensitive currents were measured at 8 different membrane potentials from –40 to +30 mV in 10 mV steps (Figures 5A,C, left) to determine the steady-state conductance-voltage relationships (Figures 5A,C, right). As previously found in type A globus pallidus or CA1 neurons (Hernandez-Pineda et al., 1999; Alshuaib et al., 2001), the TEA-sensitive currents begin to activate at ∼–20 mV (Figure 5A, right, and Figure 5B, left). Our data showed no significant difference at each of the voltage levels for steady-state conductance of both TEA-sensitive (*p* > 0.05 for all comparisons, *t*-test, Figure 5A, right) and TEA-insensitive currents (*p* > 0.05 for all comparisons, *t-*test, Figure 5C, right) in old mice. In addition, the normalized conductance (G/Gmax) for TEA-sensitive (Figure 5B, left) and TEA-insensitive currents (Figure 5D, left) allowed us to fit the data to a Boltzmann equation to obtain the half-activation voltages (V_1/2_) and activation slopes (k) of both currents. Here again, we found no difference for V_1/2_ (young vs. old, *p* > 0.05, *t*-test) and k (young vs. old, *p* > 0.05, *t*-test) of the TEA-sensitive currents (Figure 5B, right) and V_1/2_ (young vs. old, *p* > 0.05, *t*-test) and k (young vs. old, *p* > 0.05, *t*-test) of the TEA-insensitive currents (Figure 5D, right). Thus, these data indicate that there was no significant difference in the activation properties of TEA-sensitive A-type K^+^ and TEA-insensitive Kv3.1 currents, and that the ionic mechanisms underlying the observed changes in the AP and firing properties of I-S3 cells in old mice remain to be determined.

### The Inhibitory Synaptic Drive to I-S3 Cells Increases During Aging

**FIGURE 6:**
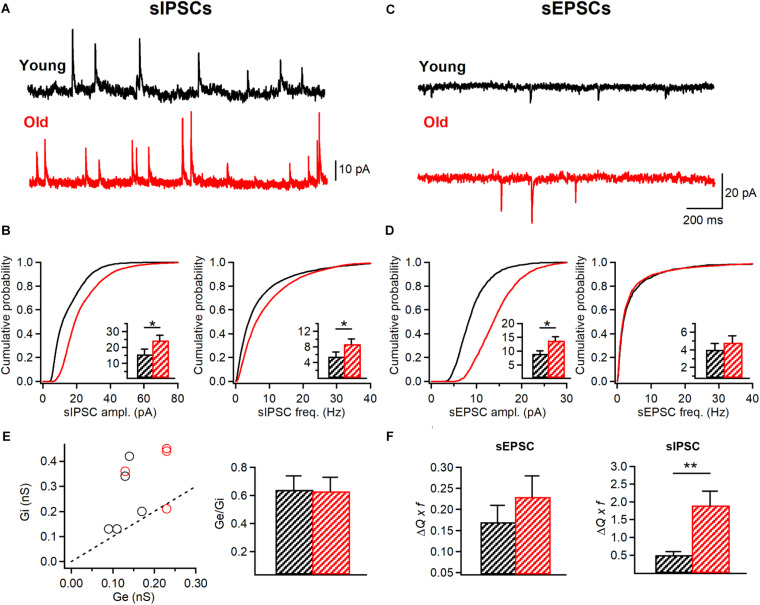
I-S3 cells receive a stronger inhibitory drive in aged mice. **(A,C)** Representative traces of sIPSCs **(A)** and sEPSCs **(C)** recorded in I-S3 cells from young (black) and old mice (red). **(B)** Summary data showing a significant increase in the amplitude and frequency of sIPSCs in I-S3 cells of aged mice (*p* < 0.001, K-S test). Insets illustrate significant differences in the sIPSC amplitude and frequency for inter-cell mean comparison (*p < 0.05, unpaired *t*-test). **(D)** Summary data showing a significant increase in the amplitude of sEPSCs (*p* < 0.001, K-S test; inset, *p* < 0.05, unpaired *t*-test) with no change in the frequency (*p* > 0.05, K-S test; inset, *p* > 0.05, unpaired *t*-test). **(E)** The inhibitory (Gi) to excitatory (Ge) conductance plot (left) illustrating a dominant inhibitory conductance in I-S3 cells across the animal lifespan. Summary data (right) for the Ge/Gi ratio in two age groups show no change in Ge/Gi in old mice due to a comparable increase in both Gi and Ge (**B,D**, left). **(F)** Summary data for the mean excitatory (left) and inhibitory (right) charge transfer when frequency of events is considered. Note a significant increase in the mean inhibitory current in I-S3 cells of old animals. *p < 0.05, **p < 0.01.

Next, to examine whether I-S3 cells could exhibit changes in their synaptic properties, we performed whole-cell voltage-clamp recordings of sIPSCs (young: *n* = 7 cells; old: *n* = 5 cells) and sEPSCs (young: *n* = 6 cells; old: *n* = 6 cells) (Figure 6). Our data revealed significant changes in both sEPSCs and sIPSCs in I-S3 cells of old mice. In particular, there was an increase in the amplitude (young: 15.6 ± 3.0 pA; old: 24.4 ± 3.3 pA, *p* < 0.001, K-S test; *p* < 0.05, *t*-test; Figures 6A,B, left) and an increase in the frequency (young: 5.5 ± 1.1 Hz; old: 8.7 ± 1.3 Hz, *p* < 0.001, K-S test; *p* < 0.05, *t*-test; Figures 6A,B, right) of sIPSCs. In parallel, the amplitude of sEPSCs was also increased (young: 9.1 ± 1.0 pA; old: 13.8 ± 1.4 pA, *p* < 0.001, K-S test; *p* < 0.05, *t-*test; Figures 6C,D, left), however, with no change frequency (young: 4.0 ± 0.7 Hz; old: 4.8 ± 0.8 Hz, *p* > 0.05, K-S test; *t*-test; Figures 6C,D, right). To determine the overall impact of these changes in sEPSCs and sIPSCs on the excitation/inhibition balance in I-S3 cells, we first calculated the inhibitory (Gi) and excitatory (Ge) conductances, and then compared these parameters and their ratio between the young and old animals. Our data showed that in both age groups, I-S3 cells receive a stronger inhibitory conductance (Figure 6E), and that the Ge/Gi ratio remains unaltered during aging due to a concomitant increase in both Gi and Ge (Figure 6E). As these parameters do not consider changes in event frequency, we next calculated a mean PSC as a product of mean PSC charge transfer and event frequency (Figure 6F). These data revealed that, while the mean EPSC remained unaltered, the mean IPSC was significantly increased due to increase in both the amplitude and frequency of sIPSCs (Figure 6B), pointing to the enhanced inhibitory drive to I-S3 cells in aged animals (Figure 6F).

### The Inhibition of I-S3 Cells Postsynaptic Targets Increases With Aging

**FIGURE 7:**
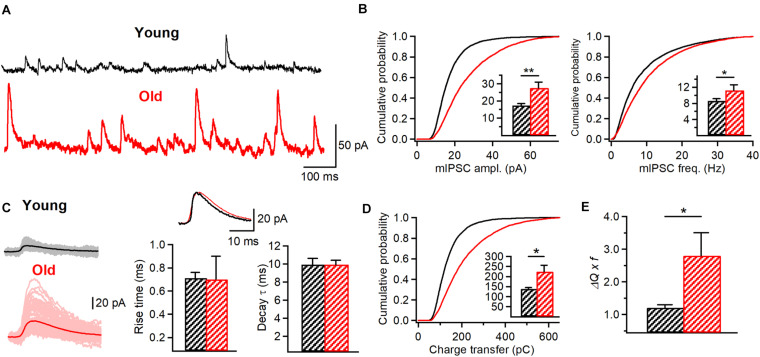
Increased inhibitory drive to O/A interneurons in aged animals. **(A)** Representative traces of mIPSCs recorded in O/A interneurons in young (black) and old (red) mice. **(B)** Cumulative probability plots of the mIPSC amplitude (left) and frequency (right) in young and old animals (K-S test, *p* < 0.001 for both plots). Insets illustrate significant differences in the mIPSC amplitude (**p < 0.01, unpaired *t*-test) and frequency (*p* < 0.05, unpaired *t*-test) for inter-cell mean comparison. **(C)** Representative examples of individual mIPSC events (100 pale sweeps, left) and average traces (bold trace, left) detected in I-S3 cells of young (black) and old animals (red). Superimposed scaled traces (right top) and summary bar graphs for a group of cells (right bottom) show no significant differences in the IPSC kinetics. **(D)** Cumulative probability plot of the mIPSC charge transfer (area under curve) in young and old animals (K-S test, *p* < 0.001). Inset illustrates significant differences (*p < 0.05, unpaired *t*-test) for inter-cell mean comparison. **(E)** Summary data for the mean inhibitory charge transfer when frequency of events is considered (*p < 0.05, unpaired *t*-test), showing a significant increase in the inhibitory drive to O/A interneurons. *p < 0.05, **p < 0.01.

The age-dependent increase in the AP duration in IS3 cells observed in our study suggests that these cells may exert a stronger impact on their postsynaptic targets via enhanced GABA release. On the other hand, an increased inhibitory drive to I-S3 cells together with a lower frequency of AP firing indicate that these cells may be less active in old mice. IS3 cells inhibit different types of O/A interneurons (Tyan et al., 2014), which, in turn, provide inhibition to different subcellular domains of CA1 PCs. To investigate wheather specific alterations in AP and firing/synaptic properties of IS3 cells could translate into a modified inhibition of O/A interneurons, we performed whole-cell voltage-clamp recordings of mIPSCs from O/A interneurons of young vs. old mice (Figure 7). Our data showed that both the amplitude (young: 17.2 ± 1.2 pA, *n* = 8; old: 27.5 ± 3.4 pA, *n* = 5, *p* < 0.001, K-S test; *p* < 0.01, *t*-test; Figures 7A,B, left) and the frequency (young: 8.6 ± 0.5 Hz, *n* = 8; old: 11.2 ± 1.4 Hz, *n* = 4, *p* < 0.001, K-S test; *p* < 0.05, *t*-test; Figures 7A,B, right) of mIPSCs recorded in O/A interneurons were significantly higher in aged animals in line with age-dependent changes at both pre- and postsynaptic sites of inhibitory synapses converging onto O/A interneurons. Furthermore, the overal charge transfer (area under curve) during mIPSC was significantly increased in O/A interneurons of old mice (young: 137.7 ± 7.1 pA x ms, *n* = 8; old: 224.2 ± 32.4 pA x ms, *n* = 5, *p* < 0.001, K-S test; *p* < 0.05, *t*-test; Figure 7C, left; Figure 7D) without significant changes in the rise time (young: 0.71 ± 0.05 ms, *n* = 8; old: 0.7 ± 0.2 ms, *n* = 5, *p* > 0.05, *t*-test; Figure 7C, right) or the decay time constant (young: 9.9 ± 0.7 ms, *n* = 8; old: 9.9 ± 0.5 ms, *n* = 5, *p* > 0.05, *t*-test; Figure 7C, right) of mIPSCs, indicating no change in the GABA_A_ receptor subunit composition. Finally, considering the increased frequency of mIPSCs we also found a significant increase in the mean IPSC charge transfer in O/A interneurons (Figure 7E). Taken together, these data reveal an age-dependent increase in inhibition of their postsynaptic targets.

## Discussion

In this study, we provide novel data on the morphological and physiological properties of a specific GABAergic interneuron subtype in aged animals. We demonstrate that VIP+/CR+ I-S3 cells survive during aging and preserve their morphological features but exhibit significant changes in the active membrane and synaptic properties, thus highlighting an age-induced functional rather than structural remodeling of this interneuron subtype. These age-dependent changes are associated with increased memory deficits as well as anxiety-like behavior reflecting pathological aging.

It is widely recognized that neuronal loss in the neocortex and hippocampus can be involved in cognitive deficits associated with aging (Morrison and Hof, 1997; Mattson and Magnus, 2006), with the dendrite-targeting SST+ interneurons such as hippocampal O-LM cells being affected the most (Stanley et al., 2012). In the current study conducted on VIP-eGFP mice, SST+ O/A interneurons were also vulnerable but VIP+/CR+ I-S3 cells remained structurally unaltered. While I-S3 cells represent less than 1% of all CA1 neurons (Bezaire and Soltesz, 2013), they are among the most excitable interneuron subtypes in the hippocampus due to a particularly high input resistance (Chamberland et al., 2010; Tyan et al., 2014). By providing behavioral state-dependent disinhibition, these cells play a critical role in spatial coding and goal-oriented reward learning (Magnin et al., 2019; Turi et al., 2019). Our data show that, in the aged hippocampus, both sEPSC and sIPSC amplitudes in I-S3 cells are significantly increased. As a result, the balance between excitatory and inhibitory conductances remains unaltered. However, inhibitory drive to I-S3 cells becomes more prominent due to a concomitant increase in sIPSC frequency and may dampen the recruitment of these cells to network activity. Interestingly, GABAergic septohippocampal innervation of CA1 CR+ interneurons is decreased during aging (Rubio et al., 2012). Thus, changes in the spontaneous inhibitory tone observed in our study may reflect increased activity of local inhibitory interneurons contacting I-S3 cells, such as other VIP+ and CR+ interneurons (Luo et al., 2020). Furthermore, while I-S3 cells receive excitatory input from the CA3 area (Luo et al., 2020), which can become hyperactive in old rodents (El-Hayek et al., 2013; Simkin et al., 2015), we have not observed any sign of hyperactivity or seizure-like excitatory patterns arriving to I-S3 cells from this region. Rather the opposite, the overall frequency of sEPSCs was very low and remained at this level during aging.

We also found an increased AP duration and steady-state depolarization in parallel with a decreased firing rate and rapid adaptation in I-S3 cells of old mice. What biophysical mechanisms could account for the observed modifications? Model simulations predicted that decreasing the fast-delayed rectifier and/or transient sodium conductances should increase the AP half-width and induce a steady-state depolarization-dependent block of firing. However, the experimental data showed no changes in the amplitude and kinetics of the fast-delayed rectifier Kv3.1 or the A-type K^+^ currents. This suggests that other subtypes of voltage-gated K^+^ channels or Na^+^ channels could be responsible for age-dependent changes in the AP and firing properties and should be examined in further studies. Moreover, recording of K^+^ currents in the soma does not discount for any possible age-dependent changes in dendritic distributions of Kv3.1 or A-type K^+^ channels. As well, age-dependent changes in currents could be occluded by variability in ion channel expression across individual cells. Importantly, increased steady-state depolarization with a prevalent rapidly adapting firing pattern of I-S3 cells in aged mice may indicate that these cells are likely to fire shorter trains of a fewer APs in response to a strong excitatory input. Although this suggests a decreased I-S3 cell output, it has been shown in CA1 PCs that full amplitude somatic APs, attenuated APs, and even small spikelets of 5–10 mV can co-occur in the axon (Apostolides et al., 2016). More specifically, somatic spikelets represent axonal APs that fail to initiate into full APs (Apostolides et al., 2016). Further, experimental and modeling data indicated that PCs APs recovered to full amplitude at more distal axonal locations as the AP-mediated K^+^ channel activation effectively counteracted the depolarizing effect of the somatic plateau (Apostolides et al., 2016). These observations indicate that I-S3 cells entering the depolarization block and having a reduction in somatic firing could still maintain the same spike frequency and train duration in axons with wider spikes leading to a longer depolarization in I-S3 axonal terminals. The tonic activation of VIP+ cells may occur during arousal and attentional tasks (Steriade, 1991) due to activation of nicotinic acetylcholine or metabotropic serotonin 5HT2 receptors (Prönneke et al., 2020) but may be impaired in aging hippocampus, with direct consequences on mnemonic processes.

Furthermore, our data show that intrinsic and synaptic remodeling of I-S3 cells may be associated with a significant increase in inhibition of their postsynaptic targets. I-S3 cells contact several distinct subtypes of O/A interneurons, including O-LM, bistratified and basket cells (Tyan et al., 2014). An increase in the AP duration in I-S3 cells during aging could indicate significant changes at I-S3 synapses, with a larger presynaptic calcium influx due to changes in Na^+^/K^+^ conductances, increased vesicular and non-vesicular GABA release, extracellular GABA accumulation and enhanced tonic inhibition through activation of extrasynaptic GABA_A_ receptors (Attwell et al., 1993; Zoli et al., 1999; Glykys and Mody, 2007), although the latter has not been detected in O-LM cells of young mice (Salesse et al., 2011). In addition, the increased inhibition of O/A interneurons reported here may be associated with a homeostatic strengthening of inhibitory synapses (Peng et al., 2010; Rannals and Kapur, 2011; Pribiag et al., 2014) following a decrease in the I-S3 input due to a higher inhibitory drive on I-S3 cells and their lower firing rate in aged hippocampus.

Importantly, aging is associated with deficits in the theta oscillation power resulting in the impaired encoding of novel spatial information in the dorsal hippocampus or emotional states in the ventral hippocampus (Jacobson et al., 2013). Modeling data combined with imaging *in vivo* of I-S3 cell activity in awake mice indicated that I-S3 cells are recruited during theta oscillations (Luo et al., 2020) and may play a role in modulating the activity of O-LM cells at theta frequency (Tyan et al., 2014). Indeed, in young animals, optogenetic activation of CR+ cells increased the post-inhibitory firing of O-LM cells specifically at theta frequency (Tyan et al., 2014). Here, we show that during aging, I-S3 cells may become less available but fire with a broader spike, resulting in the increased inhibition to O-LM cells. Whether this increased inhibition may lead to a complete suppression of O-LM cell firing remains unknown, but given a critical role of O-LM cells in theta oscillations (Gillies et al., 2002; Gloveli et al., 2005; Mikulovic et al., 2018), their silencing may have a direct impact on the frequency and power of theta rhythms as well as the encoding of novel spatial and emotion-related information. Supporting this idea, the AP broadening in I-S3 cells occurred in parallel with cognitive deficits in VIP-eGFP mice, and both variables showed a strong correlation with animal age, suggesting a causal connection.

In addition, O-LM cells play a major role in disinhibition of distal dendritic domains of CA1 PCs and facilitation of dendritic spike initiation and burst firing (Chamberland et al., 2010; Lovett-Barron et al., 2012; Müller et al., 2012; Royer et al., 2012; Tyan et al., 2014). In this regard, the increased inhibition of O-LM cells along with their decreased density in the aged hippocampus may contribute to the increased burst firing or even hyperactivity of CA1 PCs. It is to be noted that all these age-dependent modifications in the local circuit inhibition may occur in parallel with other intrinsic and synaptic changes of different cell types making up hippocampal networks. Our data reveal specific functional changes in one subtype of disinhibitory interneuron that may have an impact on the balance between hippocampal inhibition and disinhibition. However, further experiments involving cell type-specific interventions will be required to understand the mechanisms of the observed phenomena and the impact of modified disinhibitory circuits on hippocampal network activity and mnemonic processing.

## Data Availability Statement

The raw data supporting the conclusions of this article will be made available by the authors, without undue reservation, to any qualified researcher.

## Ethics Statement

The animal study was reviewed and approved by the Animal Protection Committee of Université Laval and the Canadian Council on Animal Care.

## Author Contributions

RF, SA, CH, and DT performed the experimental work. RF, AG-M, CH, FM, BM, DT, and LT analyzed the experimental data. AG-M conducted the computational modeling. RF, AG-M, and LT wrote the manuscript. RF, AG-M, M-ÈT, FS, and LT edited the manuscript. FS, M-ÈT, and LT provided resources and supervised the research. All authors contributed to the article and approved the submitted version.

## Conflict of Interest

The authors declare that the research was conducted in the absence of any commercial or financial relationships that could be construed as a potential conflict of interest.

## References

[B1] Acsády L. Arabadzisz D. Freund T. F. (1996a). Correlated morphological and neurochemical features identify different subsets of vasoactive intestinal polypeptide-immunoreactive interneurons in rat hippocampus. *Neuroscience* 73 299–315. 10.1016/0306-4522(95)00610-9 8783251

[B2] Acsády L. Görcs T. J. Freund T. F. (1996b). Different populations of vasoactive intestinal polypeptide immunoreactive interneurons are specialized to control pyramidal cells or interneurons in the hippocampus. *Neuroscience* 73 317–334. 10.1016/0306-4522(95)00609-5 8783252

[B3] Alshuaib W. B. Hasan S. M. Cherian S. P. Mathew M. V. Hasan M. Y. Fahim M. A. (2001). Reduced potassium currents in old rat CA1 hippocampal neurons. *J. Neurosci.* 63 176–184. 10.1002/1097-4547(20010115)63:2<176::aid-jnr1009>3.0.co;2-h 11169627

[B4] Apostolides P. F. Milstein A. D. Grienberger C. Bittner K. C. Magee J. C. (2016). Axonal filtering allows reliable output during dendritic plateau-driven complex spiking in CA1 neurons. *Neuron* 89 770–783. 10.1016/j.neuron.2015.12.040 26833135

[B5] Attwell D. Barbour B. Szatkowski M. (1993). Nonvesicular release of neurotransmitter. *Neuron* 11 401–407. 10.1016/0896-6273(93)90145-h 8104430

[B6] Bach M. E. Barad M. Son H. Zhuo M. Lu Y. F. Shih R. (1999). Age-related defects in spatial memory are correlated with defects in the late phase of hippocampal long-term potentiation in vitro and are attenuated by drugs that enhance the cAMP signaling pathway. *Proc. Natl. Acad. Sci. U.S.A.* 96 5280–5285. 10.1073/pnas.96.9.5280 10220457 PMC21855

[B7] Bakker A. Krauss G. L. Albert M. S. Speck C. L. Jones L. R. Stark C. E. (2012). Reduction of hippocampal hyperactivity improves cognition in amnestic mild cognitive impairment. *Neuron* 74 467–474. 10.1016/j.neuron.2012.03.023 22578498 PMC3351697

[B8] Bamidis P. D. Vivas A. B. Styliadis C. Frantzidis C. Klados M. Schlee W. (2014). A review of physical and cognitive interventions in aging. *Neurosci. Biobehav. Rev.* 44 206–220. 10.1016/j.neubiorev.2014.03.019 24705268

[B9] Barnes C. A. (1994). Normal aging: regionally specific changes in hippocampal synaptic transmission. *Trends Neurosci.* 17 13–18. 10.1016/0166-2236(94)90029-9 7511843

[B10] Bezaire M. J. Soltesz I. (2013). Quantitative assessment of CA1 local circuits: knowledge base for interneuron-pyramidal cell connectivity. *Hippocampus* 23 751–785. 10.1002/hipo.22141 23674373 PMC3775914

[B11] Bianchi D. Marasco A. Limongiello A. Marchetti C. Marie H. Tirozzi B. (2012). On the mechanisms underlying the depolarization block in the spiking dynamics of CA1 pyramidal neurons. *J. Comput. Neurosci.* 33 207–225. 10.1007/s10827-012-0383-y 22310969

[B12] Carnevale N. T. Hines M. L. (2006). *The Neuron Book.* Cambridge: Cambridge University Press 10.1017/CBO9780511541612

[B13] Chamberland S. Salesse C. Topolnik D. Topolnik L. (2010). Synapse specific inhibitory control of hippocampal feedback inhibitory circuit. *Front. Cell. Neurosci.* 4:130. 10.3389/fncel.2010.00130 21060720 PMC2972748

[B14] El-Hayek Y. H. Wu C. Ye H. Wang J. Carlen P. L. Zhang L. (2013). Hippocampal excitability is increased in aged mice. *Exp. Neurol.* 247 710–719. 10.1016/j.expneurol.2013.03.012 23510762

[B15] Fordyce D. E. Wehner J. M. (1993). Effects of aging on spatial learning and hippocampal protein kinase C in mice. *Neurobiol. Aging* 14 309–317. 10.1016/0197-4580(93)90116-S 8367012

[B16] Francavilla R. Luo X. Magnin E. Tyan L. Topolnik L. (2015). Coordination of dendritic inhibition through local disinhibitory circuits. *Front. Synaptic Neurosci.* 7:5. 10.3389/fnsyn.2015.00005 25767448 PMC4341546

[B17] Francavilla R. Villette V. Luo X. Chamberland S. Munoz E. P. Camire O. (2018). Connectivity and network state-dependent recruitment of long-range VIP-GABAergic neurons in the mouse hippocampus. *Nat. Commun.* 9:5043. 10.1038/s41467-018-07162-5 30487571 PMC6261953

[B18] Gillies M. J. Traub R. D. LeBeau F. E. N. Davies C. H. Gloveli T. Buhl E. H. (2002). A model of atropine-resistant theta oscillations in rat hippocampal area CA1. *J. Physiol.* 543 779–793. 10.1113/jphysiol.2002.024588 12231638 PMC2290530

[B19] Gloveli T. Dugladze T. Rotstein H. G. Traub R. D. Monyer H. Heinemann U. (2005). Orthogonal arrangement of rhythm-generating microcircuits in the hippocampus. *Proc. Natl. Acad. Sci. U.S.A.* 102 13295–13300. 10.1073/pnas.0506259102 16141320 PMC1201613

[B20] Glykys J. Mody I. (2007). The main source of ambient GABA responsible for tonic inhibition in the mouse hippocampus. *J. Physiol.* 582 1163–1178. 10.1113/jphysiol.2007.134460 17525114 PMC2075237

[B21] Goodrick C. L. (1975). Life-span and the inheritance of longevity of inbred mice. *J. Gerontol.* 30 257–263. 10.1093/geronj/30.3.257 1120887

[B22] Gorina Y. V. Komleva Y. K. Lopatina O. L. Volkova V. V. Chernykh A. I. Shabalova A. A. (2017). The battery of tests for experimental behavioral phenotyping of aging animals. *Adv. Gerontol.* 7 137–142. 10.1134/S2079057017020060 28557390

[B23] Guet-McCreight A. Camiré O. Topolnik L. Skinner F. K. (2016). Using a semi-automated strategy to develop multi-compartment models that predict biophysical properties of interneuron-specific 3 (is3) cells in hippocampus. *eNeuro* 3:ENEURO.0087-16.2016. 10.1523/ENEURO.0087-16.2016 27679813 PMC5035096

[B24] Guet-McCreight A. Skinner F. K. Topolnik L. (2020). Common principles in functional organization of VIP/calretinin cell-driven disinhibitory circuits across cortical areas. *Front. Neural Circuits* 14:32. 10.3389/fncir.2020.00032 32581726 PMC7296096

[B25] Gulyas A. I. Hajos N. Freund T. F. (1996). Interneurons containing calretinin are specialized to control other interneurons in the rat hippocampus. *J. Neurosci.* 16 3397–3411. 10.1523/jneurosci.16-10-03397.1996 8627375 PMC6579144

[B26] Hanks S. D. Flood D. G. (1991). Region-specific stability of dendritic extent in normal human aging and regression in Alzheimer’s disease. I. CA1 of hippocampus. *Brain Res.* 540 63–82. 10.1016/0006-8993(91)90493-f 2054634

[B27] Hernandez-Pineda R. Chow A. Amarillo Y. Moreno H. Saganich M. Vega-Saenz de Miera E. (1999). Kv3.1-Kv3.2 channels underlie a high voltage-activating component of the delayed rectifier K+ current in projecting neurons from the globus pallidus. *J. Neurophysiol.* 82 1512–1528. 10.1152/jn.1999.82.3.1512 10482766

[B28] Jacobson T. K. Howe M. D. Schimdt B. Hinman J. R. Escabi M. A. Markus E. J. (2013). Hippocampal theta, gamma, and theta-gamma coupling: effects of aging, environmental change, and cholinergic activation. *J. Neurophysiol.* 109 1852–1865. 10.1152/jn.00409.2012 23303862 PMC4868371

[B29] Koh M. T. Haberman R. P. Foti S. McCown T. J. Gallagher M. (2010). Treatment strategies targeting excess hippocampal activity benefit aged rats with cognitive impairment. *Neuropsychopharmacology* 35 1016–1025. 10.1038/npp.2009.207 20032967 PMC2820138

[B30] Lien C. C. Martina M. Schultz J. H. Ehmke H. Jonas P. (2002). Gating, modulation and subunit composition of voltage-gated K+ channels in dendritic inhibitory interneurones of rat hippocampus. *J. Physiol.* 538 405–419. 10.1113/jphysiol.2001.013066 11790809 PMC2290075

[B31] Lovett-Barron M. Turi G. F. Kaifosh P. Lee P. H. Bolze F. Sun X. H. (2012). Regulation of neuronal input transformations by tunable dendritic inhibition. *Nat. Neurosci.* 15 423–430, S1–S3. 10.1038/nn.3024 22246433

[B32] Luo X. Guet-McCreight A. Villette V. Francavilla R. Marino B. Chamberland S. (2020). Synaptic mechanisms underlying the network state-dependent recruitment of VIP-expressing interneurons in the CA1 hippocampus. *Cereb. Cortex* 30 3667–3685. 10.1093/cercor/bhz334 32080739 PMC7233006

[B33] Magnin E. Francavilla R. Amalyan S. Gervais E. David L. S. Luo X. (2019). Input-specific synaptic location and function of the α5 GABAA receptor subunit in the mouse CA1 hippocampal neurons. *J. Neurosci.* 39 788–801. 10.1523/JNEUROSCI.0567-18.2018 30523065 PMC6382980

[B34] Markham J. A. McKian K. P. Stroup T. S. Juraska J. M. (2005). Sexually dimorphic aging of dendritic morphology in CA1 of hippocampus. *Hippocampus* 15 97–103. 10.1002/hipo.20034 15390161

[B35] Mattson M. P. Magnus T. (2006). Ageing and neuronal vulnerability. *Nat. Rev. Neurosci.* 7 278–294. 10.1038/nrn1886 16552414 PMC3710114

[B36] Mikulovic S. Restrepo C. E. Siwani S. Bauer P. Pupe S. Tort A. B. L. (2018). Ventral hippocampal OLM cells control type 2 theta oscillations and response to predator odor. *Nat. Commun.* 9:3638. 10.1038/s41467-018-05907-w 30194386 PMC6128904

[B37] Morrison J. H. Hof P. R. (1997). Life and death of neurons in the aging brain. *Science* 278 412–419. 10.1126/science.278.5337.412 9334292

[B38] Moyer J. R. Disterhoft J. F. (1994). Nimodipine decreases calcium action potentials in an age- and concentration-dependent manner. *Hippocampus* 4 11–18. 10.1002/hipo.450040104 8061749

[B39] Moyer J. R. Power J. M. Thompson L. T. Disterhoft J. F. (2000). Increased excitability of aged rabbit CA1 neurons after trace eyeblink conditioning. *J. Neurosci.* 20 5476–5482. 10.1523/JNEUROSCI.20-14-05476.2000 10884331 PMC6772307

[B40] Müller C. Beck H. Coulter D. Remy S. (2012). Inhibitory control of linear and supralinear dendritic excitation in CA1 pyramidal neurons. *Neuron* 75 851–864. 10.1016/j.neuron.2012.06.025 22958825

[B41] Murman D. L. (2015). The impact of age on cognition. *Semin. Hear.* 36 111–121. 10.1055/s-0035-1555115 27516712 PMC4906299

[B42] Park J. B. Skalska S. Stern J. E. (2006). Characterization of a novel tonic gamma-aminobutyric acidA receptor-mediated inhibition in magnocellular neurosecretory neurons and its modulation by glia. *Endocrinology* 147 3746–3760. 10.1210/en.2006-0218 16675519

[B43] Peng Y. R. Zeng S. Y. Song H. L. Li M. Y. Yamada M. K. Yu X. (2010). Postsynaptic spiking homeostatically induces cell-autonomous regulation of inhibitory inputs via retrograde signaling. *J. Neurosci.* 30 16220–16231. 10.1523/JNEUROSCI.3085-10.2010 21123568 PMC6634825

[B44] Peters R. (2006). Ageing and the brain. *Postgrad. Med. J.* 82 84–88. 10.1136/pgmj.2005.036665 16461469 PMC2596698

[B45] Potapenko E. S. Biancardi V. C. Florschutz R. M. Ryu P. D. Stern J. E. (2011). Inhibitory-excitatory synaptic balance is shifted toward increased excitation in magnocellular neurosecretory cells of heart failure rats. *J. Neurophysiol.* 106 1545–1557. 10.1152/jn.00218.2011 21697450 PMC3174805

[B46] Potier B. Jouvenceau A. Epelbaum J. Dutar P. (2006). Age-related alterations of GABAergic input to CA1 pyramidal neurons and its control by nicotinic acetylcholine receptors in rat hippocampus. *Neuroscience* 142 187–201. 10.1016/j.neuroscience.2006.06.040 16890374

[B47] Potier B. Lamour Y. Dutar P. (1993). Age-related alterations in the properties of hippocampal pyramidal neurons among rat strains. *Neurobiol. Aging* 14 17–25. 10.1016/0197-4580(93)90016-5 8450929

[B48] Potier B. Rascol O. Jazat F. Lamour Y. Dutar P. (1992). Alterations in the properties of hippocampal pyramidal neurons in the aged rat. *Neuroscience* 48 793–806. 10.1016/0306-4522(92)90267-6 1630625

[B49] Power J. M. Wu W. W. Sametsky E. Oh M. M. Disterhoft J. F. (2002). Age-related enhancement of the slow outward calcium-activated potassium current in hippocampal CA1 pyramidal neurons in vitro. *J. Neurosci.* 22 7234–7243. 10.1523/JNEUROSCI.22-16-07234.2002 12177218 PMC6757904

[B50] Pribiag H. Peng H. Shah W. A. Stellwagen D. Carbonetto S. (2014). Dystroglycan mediates homeostatic synaptic plasticity at GABAergic synapses. *Proc. Natl. Acad. Sci. U.S.A.* 111 6810–6815. 10.1073/pnas.1321774111 24753587 PMC4020085

[B51] Prönneke A. Witte M. Möck M. Staiger J. F. (2020). Neuromodulation leads to a burst-tonic switch in a subset of VIP neurons in mouse primary somatosensory (barrel) cortex. *Cereb. Cortex* 30 488–504. 10.1093/cercor/bhz102 31210267

[B52] Pyapali G. K. Turner D. A. (1996). Increased dendritic extent in hippocampal CA1 neurons from aged F344 rats. *Neurobiol. Aging* 17 601–611. 10.1016/0197-4580(96)00034-6 8832635

[B53] Randall A. D. Booth C. Brown J. T. (2012). Age-related changes to Na+ channel gating contribute to modified intrinsic neuronal excitability. *Neurobiol. Aging* 33 2715–2720. 10.1016/j.neurobiolaging.2011.12.030 22284989

[B54] Rannals M. D. Kapur J. (2011). Homeostatic strengthening of inhibitory synapses is mediated by the accumulation of GABA(A) receptors. *J. Neurosci.* 31 17701–17712. 10.1523/JNEUROSCI.4476-11.2011 22131430 PMC3396123

[B55] Rapp P. R. Gallagher M. (1996). Preserved neuron number in the hippocampus of aged rats with spatial learning deficits. *Proc. Natl. Acad. Sci. U.S.A.* 93 9926–9930. 10.1073/pnas.93.18.9926 8790433 PMC38531

[B56] Rizzo V. Richman J. Puthanveettil S. V. (2014). Dissecting mechanisms of brain aging by studying the intrinsic excitability of neurons. *Front. Aging Neurosci.* 6:337. 10.3389/fnagi.2014.00337 25610394 PMC4285138

[B57] Rosenzweig E. S. Barnes C. A. (2003). Impact of aging on hippocampal function: plasticity, network dynamics, and cognition. *Prog. Neurobiol.* 69 143–179. 10.1016/s0301-0082(02)00126-0 12758108

[B58] Royer S. Zemelman B. V. Losonczy A. Kim J. Chance F. Magee J. C. (2012). Control of timing, rate and bursts of hippocampal place cells by dendritic and somatic inhibition. *Nat. Neurosci.* 15 769–775. 10.1038/nn.3077 22446878 PMC4919905

[B59] Rubio S. E. Vega-Flores G. Martinez A. Bosch C. Perez-Mediavilla A. Del Rio J. (2012). Accelerated aging of the GABAergic septohippocampal pathway and decreased hippocampal rhythms in a mouse model of Alzheimer’s disease. *FASEB J.* 26 4458–4467. 10.1096/fj.12-208413 22835830

[B60] Salesse C. Mueller C. L. Chamberland S. Topolnik L. (2011). Age-dependent remodelling of inhibitory synapses onto hippocampal CA1 oriens-lacunosum moleculare interneurons. *J. Physiol.* 589 4885–4901. 10.1113/jphysiol.2011.215244 21825029 PMC3224881

[B61] Scheibel A. B. (1979). The hippocampus: organizational patterns in health and senescence. *Mech. Ageing Dev.* 9 89–102. 10.1016/0047-6374(79)90123-4 439952

[B62] Simkin D. Hattori S. Ybarra N. Musial T. F. Buss E. W. Richter H. (2015). Aging-related hyperexcitability in CA3 pyramidal neurons is mediated by enhanced A-type K+ channel function and expression. *J. Neurosci.* 35 13206–13218. 10.1523/JNEUROSCI.0193-15.2015 26400949 PMC4579378

[B63] Somogyi P. Tamás G. Lujan R. Buhl E. H. (1998). Salient features of synaptic organisation in the cerebral cortex. *Brain Res. Rev.* 26 113–135. 10.1016/s0165-0173(97)00061-1 9651498

[B64] Stanley E. M. Fadel J. R. Mott D. D. (2012). Interneuron loss reduces dendritic inhibition and GABA release in hippocampus of aged rats. *Neurobiol. Aging* 33 431.e1–431.e13. 10.1016/j.neurobiolaging.2010.12.014 21277654 PMC3110542

[B65] Steriade M. (1991). “Alertness, quiet sleep, dreaming,” in *Cerebral Cortex*, Vol. 9 ed. Peters A. (New York, NY: Plenum Publishing Corporation), 279–357. 10.1007/978-1-4615-6622-9_8

[B66] Tucker K. R. Huertas M. A. Horn J. P. Canavier C. C. Levitan E. S. (2012). Pacemaker rate and depolarization block in nigral dopamine neurons: a somatic sodium channel balancing act. *J. Neurosci.* 32 14519–14531. 10.1523/JNEUROSCI.1251-12.2012 23077037 PMC3494994

[B67] Turi G. F. Li W. K. Chavlis S. Pandi I. O’Hare J. Priestley J. B. (2019). Vasoactive intestinal polypeptide-expressing interneurons in the hippocampus support goal-oriented spatial learning. *Neuron* 101 1150–1165.e8. 10.1016/j.neuron.2019.01.009 30713030 PMC6428605

[B68] Tyan L. Chamberland S. Magnin E. Camiré O. Francavilla R. David L. S. (2014). Dendritic inhibition provided by interneuron-specific cells controls the firing rate and timing of the hippocampal feedback inhibitory circuitry. *J. Neurosci.* 34 4534–4547. 10.1523/JNEUROSCI.3813-13.2014 24671999 PMC6608127

[B69] Wilson I. A. Gallagher M. Eichenbaum H. Tanila H. (2006). Neurocognitive aging: prior memories hinder new hippocampal encoding. *Trends Neurosci.* 29 662–670. 10.1016/j.tins.2006.10.002 17046075 PMC2614702

[B70] Wilson I. A. Ikonen S. Gallagher M. Eichenbaum H. Tanila H. (2005). Age-associated alterations of hippocampal place cells are subregion specific. *J. Neurosci.* 25 6877–6886. 10.1523/JNEUROSCI.1744-05.2005 16033897 PMC6725350

[B71] Zoli M. Jansson A. Syková E. Agnati L. F. Fuxe K. (1999). Volume transmission in the CNS and its relevance for neuropsychopharmacology. *Trends Pharmacol. Sci.* 20 142–150. 10.1016/s0165-6147(99)01343-7 10322499

